# Nano-Curcumin Inhibits Proliferation of Esophageal Adenocarcinoma Cells and Enhances the T Cell Mediated Immune Response

**DOI:** 10.3389/fonc.2013.00137

**Published:** 2013-05-29

**Authors:** Francesca Milano, Luigi Mari, Wendy van de Luijtgaarden, Kaushal Parikh, Silvia Calpe, Kausilia K. Krishnadath

**Affiliations:** ^1^Center for Experimental and Molecular Medicine, Academic Medical Center, Amsterdam, Netherlands; ^2^Department of Gastroenterology and Hepatology, Academic Medical Center, Amsterdam, Netherlands

**Keywords:** curcumin, nano-curcumin, theracurmin, esophageal adenocarcinoma, dendritic cell vaccines, T cell responses

## Abstract

In Western countries the incidence of the esophageal adenocarcinoma (EAC) has risen at a more rapid rate than that of any other malignancy. Despite intensive therapies this cancer is associated with extreme high morbidity and mortality. For this reason, novel effective therapeutic strategies are urgently required. Dendritic Cell (DC)-based immunotherapy is a promising novel treatment strategy, which combined with other anti-cancer strategies has been proven to be beneficial for cancer patients. Curcumin (diferuloylmethane), is a natural polyphenol that is known for its anti-cancer effects however, in it’s free form, curcumin has poor bioavailability. The aim of this study was to investigate whether using a highly absorptive form of curcumin, dispersed with colloidal nano-particles, named Theracurmin would be more effective against EAC cells and to analyze if this new compound affects DC-induced T cell response. As a result, we show efficient uptake of nano-curcumin by the EAC cell lines, OE33, and OE19. Moreover, nano-curcumin significantly decreased the proliferation of the EAC cells, while did not affect the normal esophageal cell line HET-1A. We also found that nano-curcumin significantly up-regulated the expression of the co-stimulatory molecule CD86 in DCs and significantly decreased the secretion of pro-inflammatory cytokines from *in vitro* activated T cells. When we combined T cells with nano-curcumin treatment in OE19 and OE33, we found that the basic levels of T cell induced cytotoxicity of 6.4 and 4.1%, increased to 15 and 13%, respectively. In conclusion, we found that nano-curcumin is effective against EAC, sensitizes EAC cells to T cell induced cytotoxicity and decreases the pro-inflammatory signals from T cells. Combining DC immunotherapy with nano-curcumin is potentially a promising approach for future treatment of EAC.

## Introduction

Esophageal adenocarcinoma (EAC) has the most rapidly increasing incidence compared to other malignancies (Gamliel, 2000; Brown et al., 2008). Overall EAC patients have a rather poor prognosis with a 5-year survival rate of <15% (Gee and Rattner, 2007; Shimada et al., 2008). Therefore, more effective treatments are urgently required. Recently, Dendritic Cell (DC)-based therapeutic cancer vaccines have proven to be a promising therapy for the treatment of cancer. For the preparation of DC-based cancer vaccines, DCs are loaded *ex vivo* with tumor antigens, and then given back to patients. After activation by the DCs, T cells become effector cytotoxic T lymphocytes (CTLs), which can recognize and lyse tumor cells (Boczkowski et al., 1996; Nair et al., 1998; Milano et al., 2007). Despite the promising advances in DC vaccination, the outcomes of patients treated with DC immunotherapy as a monotherapy are still below expectations and several critical hurdles have to be resolved to improve its effectiveness (Fox et al., 2011). It has been shown that an unfavorable tumor microenvironment, that inhibits the development and function of DCs and CTLs, plays a major role in this phenomenon (Zou, 2005, 2006). It has become clear that using DC-based therapeutic vaccines in combination with agents that modulate the tumor microenvironment, sensitize the tumor cells, or diminish the tumor bulk prior to DC treatment, would highly enhance the efficacy of this approach (Milano and Krishnadath, 2008; Kamrava et al., 2009; Dougan et al., 2010). Therefore, it is necessary to find new combinatorial approaches, which tilt the balance in favor of tumor immunity and enhance DC-induced T cell response in cancer patients. In this respect, the natural substance Curcumin 1,6-Heptadiene-3,5-dione, 1,7-bis(4-hydroxy-3-methoxyphenyl), (1E,6E)-, a derivate of the plant *Curcuma longa*, has recently gained interest. Curcumin is known to have beneficial effects against several types of cancers, such as colon, colorectal, pancreatic, and esophageal cancer (Kunnumakkara et al., 2009; O’Sullivan-Coyne et al., 2009; Sahu et al., 2009; Sandur et al., 2009; Jutooru et al., 2010). Several studies have shown that curcumin can suppress nuclear factor kappa-light-chain-enhancer of activated B cells (NF-κB) activation. Up-regulation of NF-κB is known to be a key event for carcinogenesis. Curcumin also down regulates the expression of NF-κB regulated gene products that play a role in anti-apoptosis, proliferation, invasion, angiogenesis, and metastasis (Singh and Aggarwal, 1995; Aggarwal et al., 2006; Rafiee et al., 2009; Hartojo et al., 2010). Furthermore, curcumin can down-regulate the expression of various pro-inflammatory cytokines such as TNF-α, IL-1, IL-2, IL-8, IL-12 (Xu et al., 1997; Hidaka et al., 2002; Kunnumakkara et al., 2009 Epstein et al., 2010; ). Importantly, it sensitizes pancreatic tumor cells to the chemotherapeutic drug Gemcitabine, by suppressing proliferation and angiogenesis (Kanai et al., 2010). In addition, curcumin has been proven to be remarkably safe in animal studies and in phase I/II clinical trials even in dosages as high as 12 g per day (Shankar et al., 1980; Sharma et al., 2004; Lao et al., 2006; Dhillon et al., 2008). Curcumin is classified “generally recognized as safe” (GRAS) by the US Food and Drug Administration. In spite of all the proven beneficial effects of curcumin, the major problem limiting the effect in patients, is its poor solubility in water and consequently poor bioavailability (Anand et al., 2007; Yang et al., 2007). In the present study, an effective preparation of curcumin, a nano-particle colloidal dispersion, with highly improved bioavailability and water solubility, named Theracurmin, was used (Sasaki et al., 2011). This compounds is composed of 10% curcumin, 2% other curcuminoids (demethoxycurcumin and bisdemethoxycurcumin), 46% glycerin, 4% gum ghatti, and 38% water. Because of a superior solubility in water, Theracurmin allows easier *in vitro* testing, and eventual *in vivo* administration as compared to free curcumin (Bisht et al., 2007; Anand et al., 2010). In this study, we first evaluated the direct effects of Theracurmin (nano-curcumin) on EAC cell lines. Secondly, we evaluated the direct effects of nano-curcumin on activated T cells and DCs. Finally, we tested whether nano-curcumin would sensitize the tumor cells to DC-mediated cytotoxic T cell response and would more effectively induce lysis of esophageal cancer cells.

## Materials and Methods

### Cell culture

OE19 and OE33 esophageal Barrett cancer cell lines were purchased from ECACC (Porton Down, Wiltshire, SP4 DJG, UK), and cultured in RPMI 1640 (Invitrogen, NY, USA) supplemented with 10% fetal calf serum (FCS) (Invitrogen), 100 U/ml penicillin (Invitrogen), 100 μg/ml streptomycin (Invitrogen), and 2 mmol/l l-glutamine (Invitrogen). HET-1A esophageal squamous cells were purchased from the American Type Culture Collection (Manassas, VA, USA), and cultured in MCDB-153 medium (Sigma, St. Louis, MO, USA) modified as previously described (Milano et al., 2007). All cells were cultured in a 5% CO_2_ incubator at 37 °C. The cells were maintained with twice weekly passage/refreshing medium and were harvested with trypsin-ethylenediamine tetra-acetic acid (EDTA).

### Cell treatment with nano-curcumin

Nano-curcumin (Theracurmin) was a kind gift by S. Guha (MD Anderson Cancer Center, Huston, TX, USA), and was provided by Theravalues Corporation (Tokyo, Japan). Nano-curcumin was dissolved in sterile water. After establishing the IC50 using MTS assay (data not shown), the final concentration of 50 μM nano-curcumin at the time point of 48 h was chosen. For the experiments, cells were either left untreated or exposed to 50 μM nano-curcumin for 48 h and subsequently harvested for different types of analysis.

### BrdU assay for measurement of cell proliferation

To measure cell proliferation, OE19, OE33, and HET-1A cells were plated in quadruplicate in a black 96 well microplate. After treatment with nano-curcumin, cell proliferation was measured using a BrdU incorporation assay (Roche, Almere, The Netherlands). Briefly, cells were labeled with 10 μM BrdU for 4 h at 37 °C and the labeling solution was subsequently removed. The cells were fixed and the DNA was denatured by adding FixDenat solution for 30 min at room temperature, then the anti-BrdU POD antibody was added and the plate was incubated for 90 min at RT. Next, the plate was washed 3 times and the developing substrate was added and incubated for 3 min. Finally, chemiluminescence was measured using a Synergy multi-mode microplate reader (BioTek Instruments, Winooski, VT, USA). Supernatants were collected and used to measure the effect of nano-curcumin on the cytokine production of the different cell lines by performing CBA (see description below).

### Western blot analysis

The expression of caspase-3 and procaspase-9 was detected using Western blot. After treatment with nano-curcumin, OE19, OE33, and HET-1A cells were harvested in M-PER mammalian protein extraction reagent (Thermo Scientific, Rockford, USA) and the protein concentration was determined using a BCA assay (Thermo Scientific, Rockford, USA). Equal concentrations (25 μg) of proteins were fractionated by SDS-polyacrylamide gel electrophoresis, electro-transferred to Polyvinylidene fluoride (PVDF) membranes and blocked in 5% low fat milk in TBST. For the detection of caspase-3 and caspase-9, membranes were probed using primary anti-human rabbit polyclonal caspase-3 antibody (Abcam, Cambridge, UK, ab90437) or primary anti-human rabbit polyclonal caspase-9 antibody (Santa Cruz, CA, USA, 556585). Blots were then washed with TBST and incubated for 1 h at room temperature in 1:1000 Horse Radish Peroxidase (HRP) conjugated secondary antibody in 5% low fat milk in TBST. After a final wash with TBST, blots were incubated for 5 min in Lumilight (Roche, Almere, The Netherlands) and chemiluminescence was detected using a ImageQuant LAS 4000 biomolecular imager (GE Healthcare). Band intensity from electronic images of western blots was calculated by densitometry using the public domain Java image processing program Image J (available at http://rsb.info.nih.gov/ij; developed by Wayne Rasband, National Institutes of Health, Bethesda, MD).

### Fluorescence microscopy for nano-curcumin uptake in EAC cells

Curcumin is naturally fluorescent in the visible green spectrum (Bisht et al., 2007). In order to study intracellular uptake of nano-curcumin, the cells were plated in an 8-well culture glass slide (BD Biosciences), and allowed to grow to sub-confluent levels. Thereafter, the cells were incubated with 50 μM nano-curcumin for different time points ranging from 0 to 48 h. The slides were mounted in Vectashield Mounting Medium (Vector laboratories) with DAPI (4′, 6-diamidino-2-phenylindole) in order to visualize the nucleus of the cells and visualized in the Green channel using confocal laser scanning microscope (CLSM) coupled to an inverted microscope (Olympus IX81, Japan).

### RNA isolation

RNA of the different esophageal cell lines was isolated using the RNeasy mini kit (Qiagen, Hilden, Germany). Briefly, the cells were disrupted in RLT buffer and homogenized, ethanol was added and the mixture was applied to an RNeasy mini spin column. Total RNA bonded to the membrane while contaminants were washed away using buffer RW1 and buffer RPE, containing ethanol. Finally, the total RNA was eluted in RNase-free water. Quality was determined using NanoDrop (Type ND-1000, Wilmington, USA) to measure 260/280 and 260/230 ratios. When ratios were >1.9 and >1.7, respectively, the RNA was used for further experiments.

### Dendritic cell culture and immunophenotyping

Peripheral blood mononuclear cells (PBMCs) were isolated from a fresh buffy coat (Sanquin blood bank North West, Amsterdam, The Netherlands). These were obtained from healthy volunteers that were HLA-A2 positive to obtain HLA-A2 positive PBMCs matched with HLA-A2 positive cell lines. The PBMCs were isolated by density gradient centrifugation using Ficoll. The monocytes and lymphocytes were then separated by a density separation gradient as previously described (Milano et al., 2007). The monocytes were cultured in 24 wells plates (Greiner Bio-one, Alphen aan de Rijn, The Netherlands) at a density of 5 × 10^5^ cells/ml in CellGro medium (CellGenix, Freiburg, Germany) supplemented with 800 U/ml IL-4 and 1000 IU/ml GM-CSF. At day 3, the immature DCs were stimulated for 3 days with 5 μg/ml monophosphoryl lipid A (MPLA) (Invivogen, San Diego, USA) and 1000 IE/ml IFN-γ, to obtain mature DCs, which were harvested and used for stimulation of T cells as described before (ten Brinke et al., 2007). Mature DCs were then incubated with 0 or 50 μM nano-curcumin, and then harvested after 48 h to evaluate the effect of nano-curcumin on their immunophenotype by FACS analysis as described previously (Milano et al., 2007). Briefly, DCs were washed and incubated with primary anti-human antibody for CD80, CD86, and CCR7 or isotype control in PBA (PBS containing 0.5% sodium azide). After 30 min of incubation on ice in the dark, the cells were washed, re-suspended in PBA and analyzed on a FACSCalibur (BD Biosciences). The data were analyzed by using FlowJo software (Tree Star, Inc., Ashland, OR, USA). Supernatants were as well collected to measure the effect of nano-curcumin on cytokine levels performing CBA, as described below.

### T cell cultures and immunophenotyping

T lymphocytes were isolated from PBMC obtained from buffy coats. PBMC were isolated by centrifugation on Ficoll (GE Healthcare Bio-Sciences) and the T cell fraction was immediately cryopreserved. On the day of the experiment, T cells were thawed and cultured in RPMI 1640 (Invitrogen, NY, USA) supplemented with 10% FCS (Invitrogen). The T cell Activation/Expansion kit (Miltenyi Biotec, Bergisch Gladbach, Germany) was used to activate the T cells. Briefly, cells were incubated with anti-biotin MACSiBead particles loaded with CD2, CD3, and CD28 antibodies, using one loaded anti-biotin particle per two T cells, for 48 h at 37 °C. Resting or activated T cells were incubated with 0 or 50 μM nano-curcumin, and then harvested after 48 h to evaluate the effect of nano-curcumin on their immunophenotype by FACS analysis as previously described (Milano et al., 2007). Briefly, either resting or activated T cells were washed and incubated with primary anti-human antibody for CD4, CD8, or isotype control in PBA (PBS containing 0.5% sodium azide). After 30 min of incubation on ice in the dark, the cells were washed, re-suspended in PBA and analyzed on a FACSCalibur (BD Biosciences). The data were analyzed by using FlowJo software (Tree Star, Inc., Ashland, OR, USA). Supernatants were as well collected to measure the effect of nano-curcumin on cytokine levels performing CBA, as described below.

### Transfection of DC with tumor RNA and co-incubation with T cells

Mature DCs were harvested at day 6 of culture and after washing they were electroporated using the Amaxa cell line Nucleofector Kit V (Amaxa GmbH, Germany). DCs were mixed with Nucleofector transfection solution V and 4 μg of total RNA of OE19 or of OE33 or HET-1A cells. The program U16 of the Amaxa transfection device was used to electroporate the cells. After this, electroporated DCs were co-cultured with T lymphocytes at a ratio of 1:4 in a 24 wells plate. After 1 week the T cells were harvested and again co-cultured with freshly electroporated DCs for a second stimulation. The different populations of T cells, namely CTLs specific for the different cell line antigens, were used in the cytotoxicity assay to determine their killing capacity against EAC cancer cell lines.

### Effect of DC-mediated T cell responses and nano-curcumin treatment on cell lysis of tumor cells

The OE19, OE33, and HET-1A cell lines were pre-treated with 0 or 50 μM nano-curcumin for 48 h. Subsequently, 10,000 target cells were harvested, washed, and co-incubated with different amount of effector cells i.e., the above mentioned HLA-matched specific CTLs that were stimulated with tumor RNA electroporated DCs. DC and CTLs were co-cultured at an effector:target ratio of 10:1 to 0,625:1 in 100 μl of medium in a 96 wells plate for 4 h at 37 °C. The percentage of cytotoxicity was measured by using CytoTox 96 Non-Radioactive Cytotoxicity assay (Promega, Madison, USA) following the manufacturer’s instructions and as previously described (Milano et al., 2010). This assay quantitatively measures lactate dehydrogenase (LDH), which is a stable cytosolic enzyme released upon cell lysis. The released LDH in culture supernatants is measured with a 30-min coupled enzymatic assay, which results in the conversion of a tetrazolium salt (INT) into a red formazan product. The amount of color is proportional to the number of lysed cells. Visible wavelength absorbance data was collected using a multi-well scanning spectrophotometer (ELISA reader). The percentage of specific cytotoxicity was calculated using the formula: % cytotoxicity = (Experimental − Effector Spontaneous − Target Spontaneous)/(Target Maximum − Target Spontaneous) × 100.

### CBA assay

Supernatant collected from the cytotoxicity assays, and from the cultures of DC, T cells and the EAC cell lines, untreated or treated with nano-curcumin were analyzed for the simultaneous measurement of different pro- and anti-inflammatory cytokines using the cytometric bead enzyme-linked immunosorbent assay systems (CBA, BD Biosciences). Specifically, the Human Th1/Th2 cytokines kit and the Human Inflammatory kit were used according to the manufacturer’s protocol, as previously described (Milano et al., 2008).

### Statistics

Data is represented as mean ± SD. Comparison between groups was carried out with Student’s *t* test. Values of *P* < 0.05 were considered as statistically significant. Asterisks indicate the level of significance. All the experiments were carried out at least 3 times (*n* = 3) with 2–4 technical replicate.

## Results

### Nano-curcumin uptake assay in esophageal adenocarcinoma cell lines

**Figure 1:**
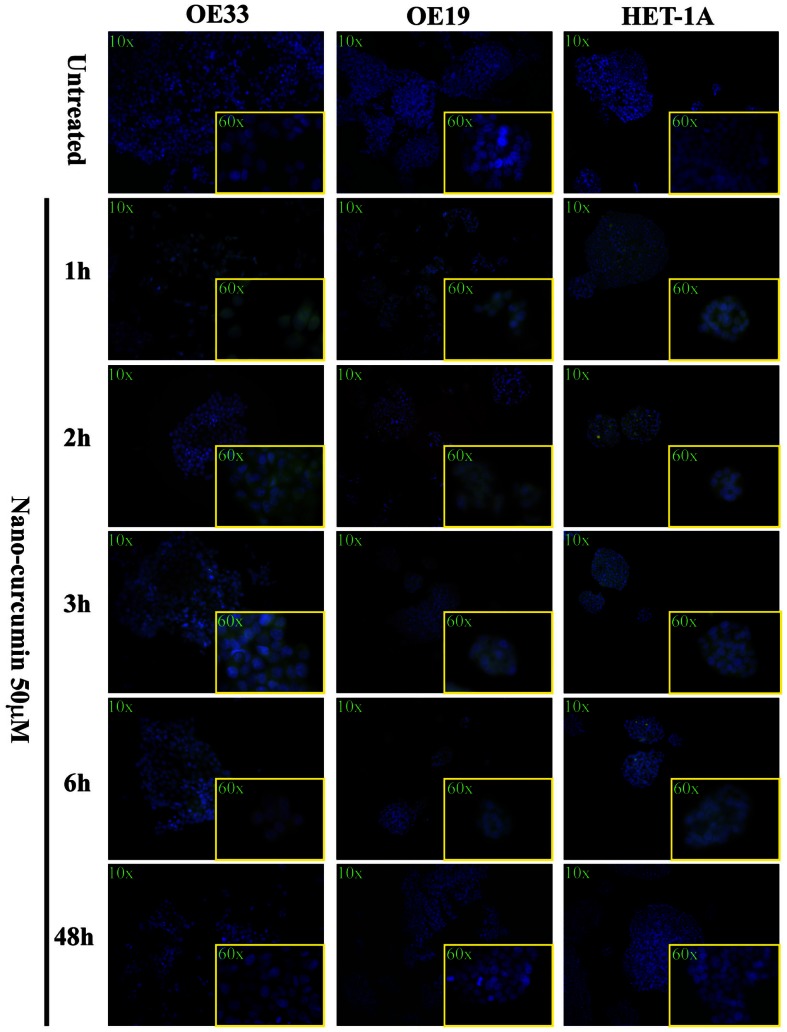
**Uptake of nano-curcumin in EAC cell lines compared to normal esophageal cell lines**. Fluorescence microscopy analysis shows that nano-curcumin, which is green autofluorescent is rapidly (within 1 h) taken up by cells. The uptake seems to increase with time and it is still observed after 6 h. Micrographic pictures are representative of three independent experiments.

To confirm that the structural changes in the preparation of this nano-curcumin, do not affect its cellular uptake in esophageal cell lines, we monitored its intracellular accumulation using CLSM coupled to an inverted microscope (Olympus IX81, Japan). In Figure 1 it is shown that after 1 h of incubation with 50 μM nano-curcumin, all the cell lines show a green signal, which increased after 2 and 4 h, as compared to the negative untreated cells. At 6 h the fluorescent signal of nano-curcumin decrease and at 48 h it could not be visualized anymore by fluorescent microscopy. Our results are in line with previous findings, where it was shown that once taken up by cells, nano-curcumin is rapidly (within 7 h) metabolized and becomes invisible after 48 h (Bisht et al., 2007; Kunwar et al., 2008; Mathew et al., 2012). No major differences were observed between the esophageal cancer (OE33 and OE19) and normal (HET-1A) cell lines.

### Effect of nano-curcumin on cell proliferation of EAC cell lines

**Figure 2:**
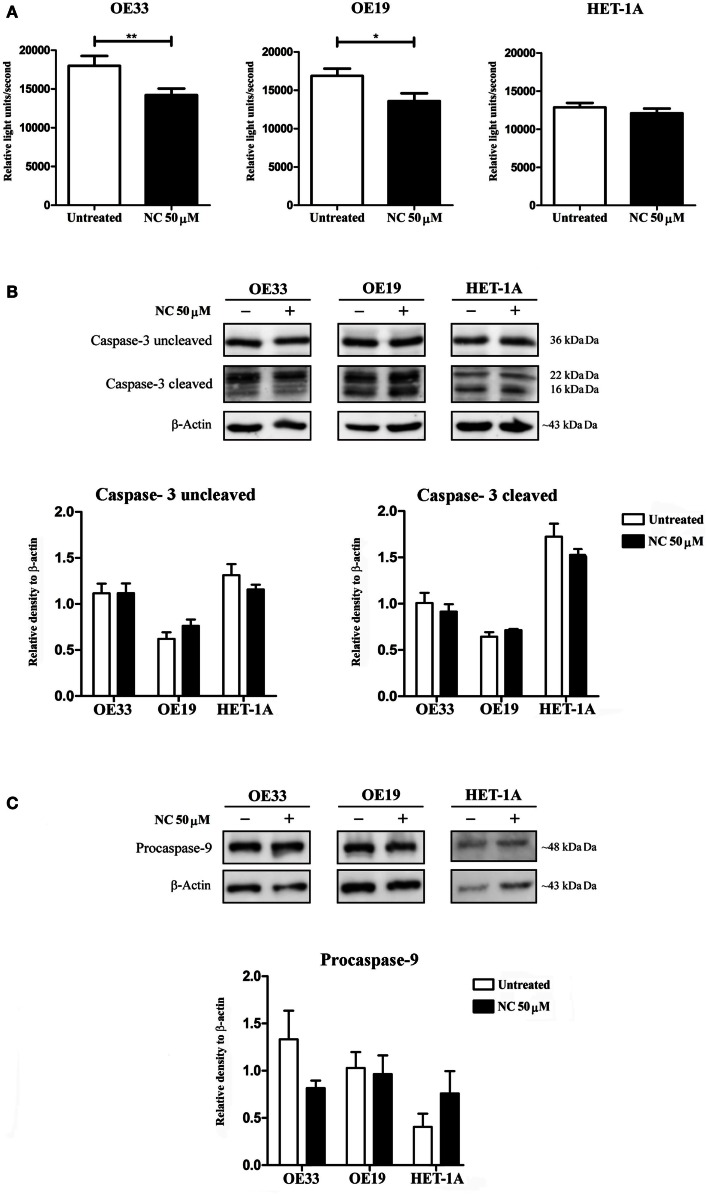
**Nano-curcumin inhibits cell proliferation and does not induce apoptosis in EAC cell lines**. **(A)** BrdU assay results show that treatment of the EAC cell lines OE33 and OE19 with 50 μM nano-curcumin (NC) induces a significant decrease in cell proliferation. This is not the case for the normal esophageal cell line HET-1A (student’s two tailed paired *t* test, *P < 0.05, **P < 0.01, *n* = 3). **(B,C)** Western blot analysis to check levels of cleaved caspase-3 and procaspase-9 shows that treatment of the EAC cell lines OE33 and OE19 with 50 μM nano-curcumin does not affect the levels of cleaved caspase-3 and procaspase-9. This is as well shown by densitometry data showing the ratio of cleaved caspase-3 and procaspase-9 to the loading control β-actin using Image J software (student’s two tailed paired *t* test, *n* = 3). Gels are representative of three independent experiments.

The functional effect of nano-curcumin on cell proliferation of the EAC cell lines, OE19, OE33, and the normal squamous cells, HET-1A, was investigated using a BrdU incorporation assay. Figure 2A shows that treatment of the cells with 50 μM nano-curcumin for 48 h, significantly decreased cell proliferation in the EAC cell lines OE19 and OE33, but not in the normal esophageal squamous cell line HET-1A, indicating that nano-curcumin selectively affects the proliferation of cancer cells leaving the normal squamous epithelial cells unaffected.

### Effect of nano-curcumin on apoptosis of EAC cell lines

To test whether the inhibition of proliferation correspond to an increase in apoptosis, we set out to investigate if the apoptotic signaling pathways are affected after treatment with nano-curcumin. By performing Western blot on the lysates of the cell lines, we could not detect a significant up-regulation of cleaved caspase-3 (the activated form of caspase-3) nor a significant decreasing levels of procaspase-9 after treatment with nano-curcumin, as shown by the densitometry of the western blots indicating that it does not induce apoptosis in these cell lines (Figures 2B,C). Levels of several other pro- and anti-apoptotic proteins and a Nicoletti apoptosis assay confirmed these findings (data not shown).

### Effect of nano-curcumin on the immunophenotype, cytokine production and apoptosis of DCs and T cells

**Figure 3:**
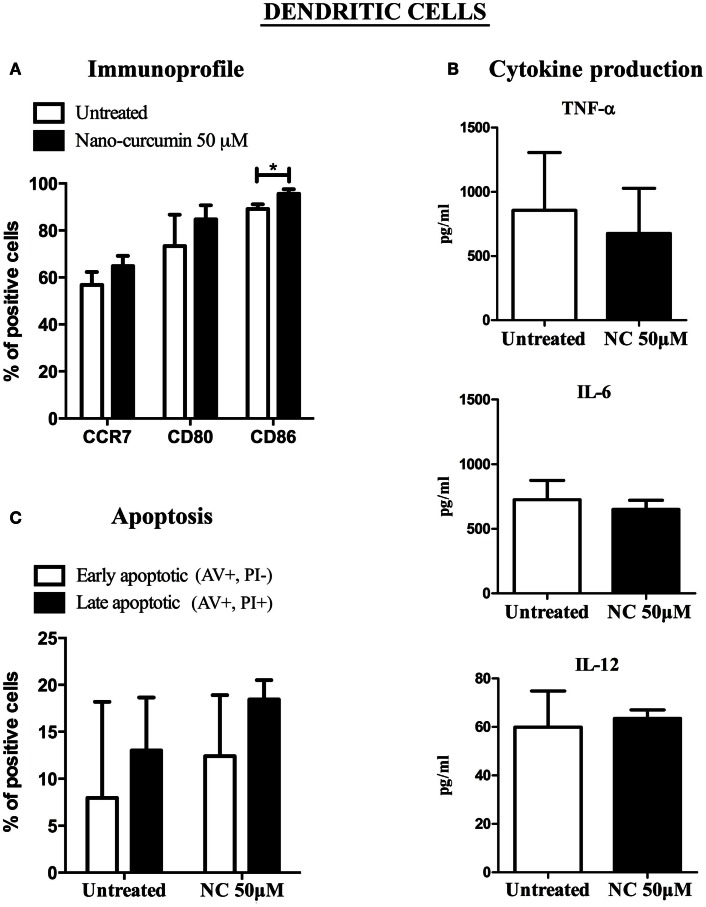
**Nano-curcumin does not interfere with the function of dendritic cells (DC), and increases expression of the co-stimulatory molecule CD86**. **(A)** Fluorescence activated cell sorting (FACS) analysis shows that nano-curcumin does not change the expression of CD80 and CCR7 in DC, but it does significantly increase the expression of CD86 on DCs (student’s two tailed paired *t* test, *P < 0.05, *n* = 4). **(B)** Cytometric bead array show that nano-curcumin does not change the cytokine production profile of the DCs (student’s two tailed paired *t* test, *n* = 3). **(C)** Flow cytometric detection of annexin V (AV) and propidium iodide (PI) shows that nano-curcumin does not induce apoptosis in DCs. Data are showing the percentage of early apoptotic (annexin V+/PI−) and late apoptotic/necrotic (annexin V+/PI+) populations (student’s two tailed paired *t* test, *n* = 3).

In previous studies it was shown that curcumin negatively affects the immunophenotype of DCs (Kim et al., 2005). To determine whether nano-curcumin has detrimental effects on cells of the immune system, we first studied the changes in the immunophenotype, cytokine profile and apoptosis level of DCs before and after exposure to nano-curcumin. Expression levels of CD80 and CCR7 in DCs did not change before and after exposure to nano-curcumin. Thus unlike previous reports on free curcumin, nano-curcumin leaves the functional phenotype of DCs intact (Figure 3A). Also, we observed that nano-curcumin significantly increased the expression level of the co-stimulatory molecule CD86 in DCs, indicating that it drives DCs to mature toward a functional phenotype. We also observed that nano-curcumin has no effects on the cytokine profile of these DCs (Figure 3B) and that it did not induce apoptosis of DCs (Figure 3C).

**Figure 4:**
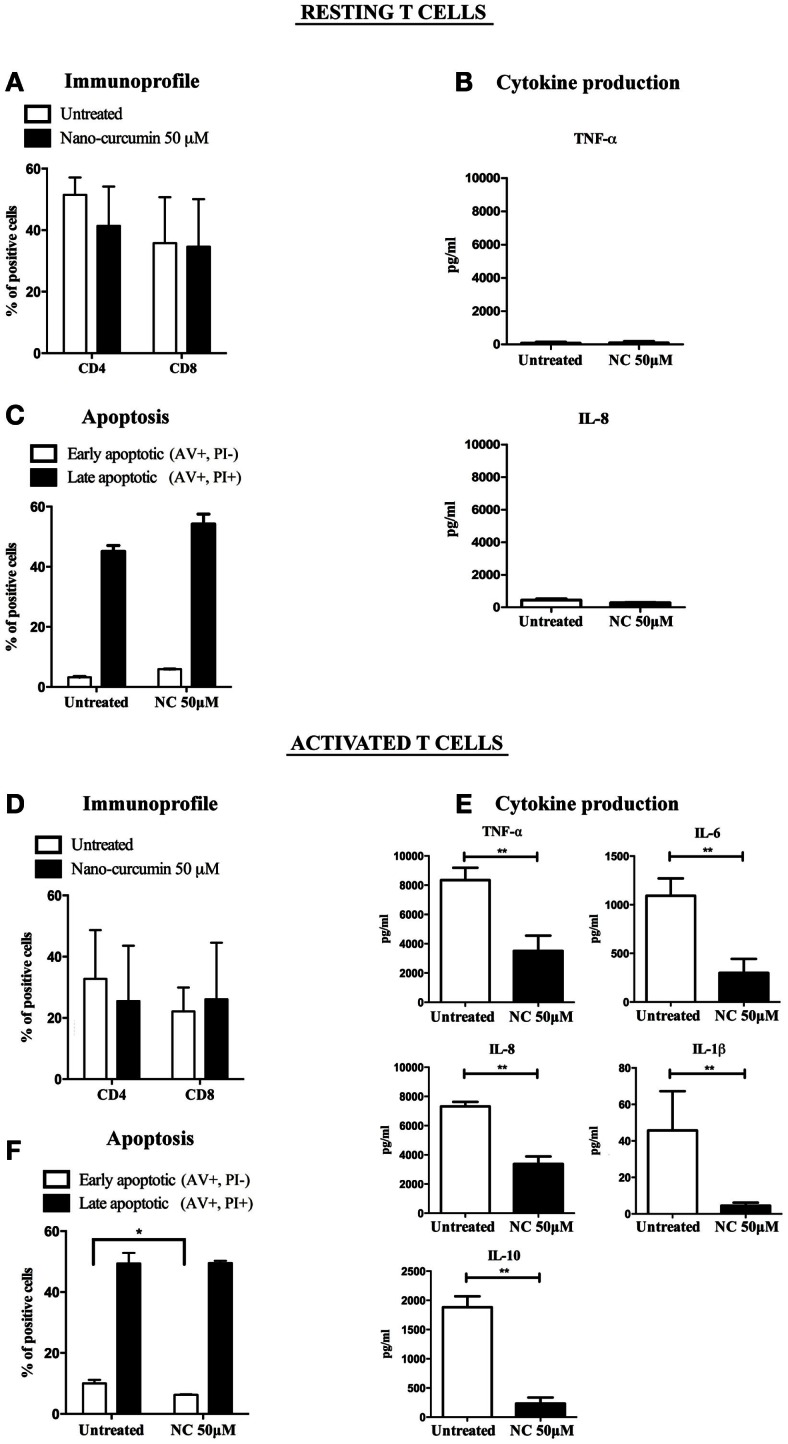
**Effect of nano-curcumin on resting and activated T cells**. **(A)** As measured by FACS, the CD4/CD8 ratio of resting T cells does not change after nano-curcumin treatment (student’s two tailed paired *t* test, *P* > 0,05, *n* = 3). **(B)** Nano-curcumin does not affect the release of TNF-α and IL-8 of resting T cells (student’s two tailed paired *t* test, *P* > 0,05, *n* = 3). **(C)** The fractions of early apoptotic (annexin V+/PI−) and late apoptotic/necrotic (annexin V+/PI+) populations as measured by FACS analysis show no difference in apoptosis between resting T cells that were left untreated or treated with nano-curcumin (student’s two tailed paired *t* test, *P < 0.05, *n* = 3). **(D)** As measured by FACS, the CD4/CD8 ratio of activated T cells does not change after nano-curcumin treatment (student’s two tailed paired *t* test, *P* > 0,05, *n* = 3). **(E)** Nano-curcumin reduces the production of TNF-α, IL-8, IL-6, IL-10, IL-1β in activated T cells (student’s two tailed paired *t* test, **P < 0.01, *n* = 3). **(F)** There is a decrease of the early apoptotic (AV+/PI−) fraction of the activated T cells after treatment with nano-curcumin (student’s two tailed paired *t* test, *P < 0.05, *n* = 3).

Next, we investigated the effect of nano-curcumin on T cell phenotype and function. We showed that nano-curcumin did not change the phenotype of resting nor activated T cells as observed by unchanged expression levels of CD4 and CD8 after treatment (Figures 4A,D). In activated T cells, nano-curcumin significantly decreased the amount of early apoptotic cells (Figure 4F) but had no effect on resting T cells (Figure 4C). Accordingly, nano-curcumin did not affect the production of cytokines of resting T cells (Figure 4B), but it did significantly reduce the secretion of TNF-α, IL-8, IL-6, IL-10, and IL-1β in activated T cells. This indicates that nano-curcumin modulates the cytokine profile of activated T cells toward a profile that negatively affects tumor cell growth and migration (Figure 4E).

From our results we can conclude that nano-curcumin has no negative effects on the immune-profile of both DCs and T cells and does not negatively affect their function. Instead, these results for the first time show that nano-curcumin supports the function of DCs and T cells in inducing anti-tumor immune responses.

### Effect of nano-curcumin on DC-mediated T cell-induced cytotoxicity

**Figure 5:**
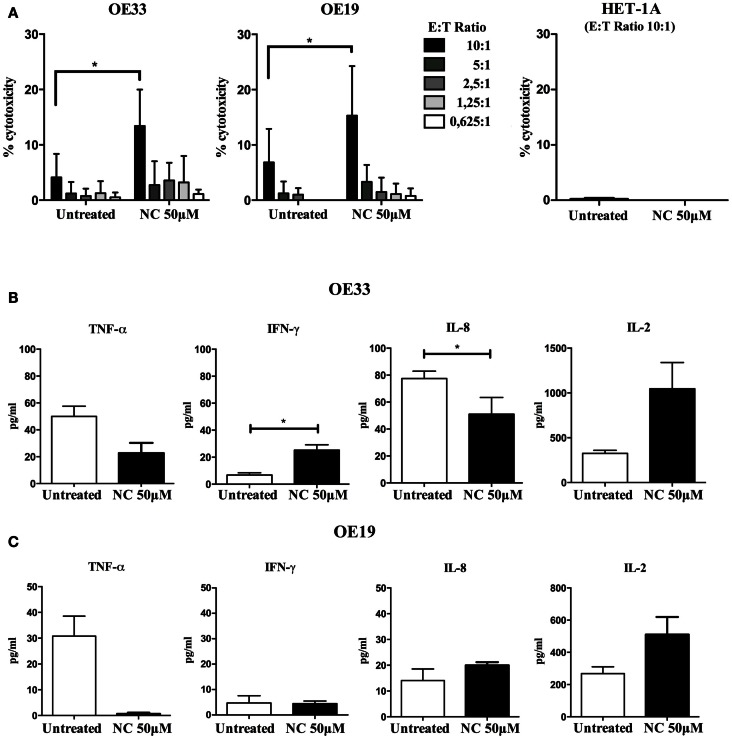
**Nano-curcumin enhances T cell mediated cancer cell lysis**. **(A)** Cytotoxicity assay showing different E:T ratios of OE19 and OE33 co-culture with CTLs. Cell lysis induced by CTLs at the E:T ratio of 10:1 is 6.4 and 4.06%, respectively. Pre-treatment with nano-curcumin, significantly increases the CTLs induced lysis to 15 and 13% respectively (E = effector = CTLs, T = target = EAC cells; student’s two tailed paired *t* test, *P < 0.05, *n* = 3). At the E:T ratio of 10:1 there is no cytotoxicity induced by CTLs on the HET-1A cells, which also does not change after nano-curcumin treatment. **(B)** The cytokine profile as measured in the supernatant after the cytotoxicity assay shows that in the co-culture of CTLs and OE33, pre-treatment with nano-curcumin increases IFN-γ production and decreases the production of IL-8 (student’s two tailed paired *t* test, *P < 0.05, *n* = 3). **(C)** In the supernatant of the co-culture of OE19 cells with CTLs, pre-treatment with nano-curcumin did not change the cytokine profile (student’s two tailed paired *t* test, *n* = 3).

To determine whether nano-curcumin enhances DC-mediated T cell induced cytotoxicity, we tested the effects of nano-curcumin pre-treatment of EAC cell lines in CTL cytotoxicity assays. Through electroporation DCs were loaded with the RNA of OE19 and OE33 cell lines. Specific anti-cancer CTL populations were obtained through stimulating T cells with the RNA loaded DCs. Using an effector (CTLs) to target (EAC cells) ratio of 10:1, in OE19 and OE33 cells, the CTLs induced a mean cell lysis of 6.4 and 4.06%, respectively (Figure 5A). Pre-treatment of the tumor cells with 50 μM nano-curcumin significantly increased the mean cell lysis to 15 and 13%, respectively. This indicates that pre-treatment with nano-curcumin sensitizes EAC cells to specific CTL-induced cytotoxicity. In a similar experiment we found no cytotoxicity against the normal esophageal cell line HET-1A (Figure 5A). We also evaluated the production of cytokines after 4 h of incubation of the EAC target cells with the effector cytotoxic T cells with and without pre-treatment of nano-curcumin. We found that in the co-culture of OE19 with CTLs there were no changes in the cytokine production profile (Figure 5C). When CTLs where incubated with OE33, however, nano-curcumin pre-treatment significantly increased the production of IFN-γ, while the production of IL-8 significantly decreased. No significant changes were observed for TNF-α and IL-2 (Figure 5B). This is interesting considering that IL-8 was found to be highly expressed in OE33 and OE19 (data not shown) and was previously reported to be highly expressed in EAC (Milano et al., 2008). Reduced levels of IL-8 may reduce the migratory functions of esophageal cancer cells, while higher levels of IFN-γ support the function of DCs and T cells, again indicating that nano-curcumin seems to enhance the function of the immune system against tumor cells.

## Discussion

Curcumin is a natural substance that is known to have anti-carcinogenic and anti-inflammatory effects against several types of cancers (Kunnumakkara et al., 2009; O’Sullivan-Coyne et al., 2009; Jutooru et al., 2010; Yallapu et al., 2012). A disadvantage of free curcumin is that it is highly hydrophobic and is poorly absorbed after oral administration (Li et al., 2005). Due to this low biological activity, high doses of free curcumin are necessary to obtain significant responses (Bhawana et al., 2011). Various curcumin nano-formulations have been developed to improve its solubility, bioavailability, and pharmacokinetic properties, allowing easier *in vitro* and preclinical *in vivo* testing. Sasaki et al. (2011), recently formulated an innovative colloid-based preparation of curcumin named Theracurmin (nano-curcumin) and demonstrated its oral bioavailability and safety in healthy subjects. The toxicity of this type of nano-curcumin is currently being tested in a clinical trial in patients with advanced malignancies (ClinicalTrials.gov identifier: NCT01201694). So far, besides one single event of diarrhea, no adverse events have been recorded, again indicating that this form of nano-curcumin is safe and well tolerated (Kanai et al., 2012).

Although these studies deem nano-curcumin as a safe substance, its biological function, has yet to be confirmed. Because other forms of nano-curcumin have the same biological effects as free curcumin in pancreatic and prostate cancer (Bisht et al., 2007; Yallapu et al., 2012), we set out to demonstrate that nano-curcumin retains the anti-carcinogenic and anti-inflammatory properties attributed to free curcumin, and thus be used as a possible adjuvant for the treatment of EAC.

We show that nano-curcumin has a direct anti-proliferative effect on EAC cell lines, in line with previous results showing that free curcumin decreases the proliferation and survival of esophageal cancer cells (Subramaniam et al., 2012). It is worth mentioning the specific anti-proliferative effect of nano-curcumin toward cancer but not normal cell lines. This characteristic is not due to differential cellular uptake, as we have proved that intracellular accumulation of nano-curcumin is equal in both types of cell lines. Instead, it could be speculated that divergences in signaling pathways between cancer and normal cells account for the selective proliferative effects of the nano-curcumin. For example, the signaling pathways affected by nano-curcumin, might be more activated in esophageal cancer cells as compared to normal cells, rendering the cancer cells more susceptible to the effects of nano-curcumin. Indeed, one of the most important pathways involved in cellular proliferation and aberrantly activated in cancer stem cells, the Notch signaling pathway, has been shown to be inhibited by free curcumin in esophageal cell lines (Subramaniam et al., 2012). Therefore, it is tempting to speculate that by inhibiting this pathway, nano-curcumin might selectively suppress proliferation of the EAC cell lines.

Although nano-curcumin has been demonstrated to also induce apoptosis in a variety of cells including pancreatic cancer cells (Sahu et al., 2009; Jutooru et al., 2010), we did not observe an effect on apoptosis of esophageal cell lines. Several mechanisms for curcumin-mediated apoptosis have been suggested. Shankar et al. (2007), for instance demonstrated that curcumin upregulates the expression of pro-apoptotic members of the Bcl-2 family like Bax and Bak and inhibits the anti-apoptotic Bcl-2 proteins, such as Bcl-X_L_ and Bcl-2. Also curcumin was found to affect several caspases such as caspase-8 (Anto et al., 2002). We tested the levels of the above mentioned pro- and anti-apoptotic pathways, including Bcl-2 and Bcl-X_L_s, but could not see any significant change in any of the apoptotic pathways after treating the EAC cells with nano-curcumin. The discrepancy between our results and the above mentioned studies could be attributed to differences in signaling pathways between the different cancer cells or to the intrinsic resistance of EAC cells to apoptosis, as it has been previously reported (O’Sullivan-Coyne et al., 2009).

Another level at which Theracurmin exerts its anti-carcinogenic effect on esophageal tumor cells is by increasing their susceptibility to be killed by cytotoxic T cells. CTLs were stimulated *ex vivo* with DCs loaded with tumor-derived RNA, and were used in cytotoxic assays to determine their ability to recognize and lyse EAC cells. We found that nano-curcumin has a sensitizing effect on DC-mediated T cell cytotoxicity by increasing cell lysis on EAC cells. We also observed that nano-curcumin increased the IFN-γ secretion and decreased the TNF-α secretion in the co-culture of OE33 with CTLs and nano-curcumin.

One earlier report showed that curcumin has a detrimental effect on the immune-phenotype of DCs (Kim et al., 2005). In our study, however, we found that neither DCs nor T cells are negatively affected by nano-curcumin. Instead, we found that nano-curcumin up-regulated the expression of the co-stimulatory molecule CD86 and reduced the levels of anti-inflammatory cytokines in activated T cells, asserting the role of nano-curcumin in anti-inflammatory processes. This is as well in line with previous findings were it was shown that nano-curcumin reduces the levels of multiple pro-inflammatory cytokines (Bisht et al., 2007; Anand et al., 2010).

It has become clear that combining anti-cancer treatments potentiates the effect of anti-cancer agents (Vanneman and Dranoff, 2012). For instance, the combination of curcumin with Gemcitabine for the treatment of pancreatic cancer, leads to increased apoptosis *in vitro* and reduced cell proliferation *in vivo* (Buckanovich et al., 2008; Dhillon et al., 2008).

One important direction in the field of oncology is to combine conventional (chemo) therapeutical agents with strategies that enhance the immune system (Ramakrishnan et al., 2010). Our results confirm that nano-curcumin not only directly affects EAC cancer cell proliferation but also potentiates the immune response to the tumor cells, making this compound extremely attractive to be used in immune combinatorial therapies for EAC.

However, further *in vivo* evaluations are warranted to confirm its efficacy as a novel and more efficacious adjuvant therapy for this aggressive cancer.

## Conflict of Interest Statement

The authors declare that the research was conducted in the absence of any commercial or financial relationships that could be construed as a potential conflict of interest.
